# Registered nurses’ perceptions on the factors affecting nursing shortage in the Republic of Vanuatu Hospitals: A qualitative study

**DOI:** 10.1371/journal.pone.0251890

**Published:** 2021-05-20

**Authors:** Adel Tutuo Tamata, Masoud Mohammadnezhad, Ledua Tamani

**Affiliations:** 1 Vanuatu College of Nursing Education, Ministry of Health, Port Vila, Vanuatu; 2 School of Public Health and Primary Care, Fiji National University, Suva, Fiji; International Medical University, MALAYSIA

## Abstract

**Background:**

Registered nurse has a vital role in delivering healthcare services to individual, family and community. One of the main challenges that health system facing globally is the shortage of nursing workforce. Vanuatu as a Pacific county is also facing the shortage issue and the impact on the registered nurses’ performance.

**Methods:**

A qualitative study was used to collect data from 25 registered nurses in three randomly selected hospitals in Vanuatu between 4^th^ to 14^th^ September, 2020. A semi-structured open-ended questionnaire was used to collect data using face-to-face in-depth interviews. The data were transcribed and analyzed using thematic analysis process.

**Results:**

Four themes were identified including; Difficult working conditions, Reinforcing factors and Perceived risks. Sub themes for difficult working condition were heavy workload, lack of workforce and unusual working hours. Sub themes for reinforcing factors were lack of support, lack of opportunities and advancement in nursing practice. Sub themes for perceived risks were stress, physical and mental risk, and social and family risks.

**Conclusion:**

This study has identify factors affected shortage of current nursing workforce and the impact it has on registered nurses. Broad themes and sub-themes were identified which highlighted the impact of nursing shortage to registered nurses and the effects on their performance which includes stress or moral distress from work overload and lengthy hours shift which impact the nurses’ physical, psychological, social, and family relationship, and lack of leadership support. The findings can be helpful to policy makers at the decision-making level to resolve the nursing workforce shortage and its effects in the future by refining and developing relevant policies that will address and strengthen the nursing workforce to meet the demand and improve delivery of quality health services to all individual.

## Introduction

Registered Nurses (RNs) are valued professionals and constitute the largest proportion of nursing population. They play a very significant role to ensure that effective quality care is provided in improving the health system [1]. In order to improve the health coverage and achievements of health targets, adequate nurses are crucial as the effectiveness of the patient care depend on the availability of more nurses [2, 3].

While the world has acknowledged nursing profession as vital in delivering healthcare services, one of the main challenges faced today globally is the shortage of nursing workforce which has major impact on nurses and causes severe effects on the nurses’ performance to provide quality of health care services and improving well-being of the global population [3–5]. The nursing shortage caused severe stress or burned out which aggravate the problems on nurses to leave their job [1].

According to the World Health Organization (WHO), it was estimated that there will be a shortage of 7.2 million health workers to deliver healthcare services worldwide and by 2035 the demand of nursing will reach 12.9 million [6]. The inadequate supply of nurses has notably created many negative impacts not only on RNs but also on patient health-related outcome as well as challenges to fight diseases and improving health, which causes increase workload on nurses and later results in decreasing the quality of nursing care [7, 8].

There are many factors affecting the healthcare system as a result of shortage of nursing workforce. These include decreased number of student nurse’s enrolment in nursing program and increase number of early retirement due to health problem [3, 9]. However, one of the main factors reported in many countries is inadequate policies and workforce planning [10, 11].

In the Pacific Island Countries (PICs), the shortage in nursing workforce is becoming a common problem [9]. In Solomon Islands, Papua New Guinea and Vanuatu, the health worker density per 1,000 populations (mainly nurses and midwives) is far below the minimum threshold density (4.45 per 1,000 populations) to sustain basic health services [12]. In countries such as Tonga, Samoa and Fiji, the main factors that trigger shortage of nursing staff includes very high rate of nurses’ migration to other countries, especially to Australia and New Zealand for better working conditions and for other potential opportunities. This has created challenges and gaps that needed to be identified to better explore the extent of the nursing shortage and to address it promptly and efficiently [13].

In Vanuatu, nurses constitute only 58% or 12.0 per 10,000 populations, which is below the WHO recommended ratio of 45 nurses per 10,000 populations [14]. According to the Vanuatu Ministry of Health (MoH) Annual Report (2018), the number of retiree nurses in the next 10 years will continue to rise but will be disproportionate to the qualified nurses graduated from the Vanuatu College of Nursing Education (VCNE) which becoming a major problem for Vanuatu MoH to fill the vacant positions. This will create more workload for nurses which will impact their performance. This study sets out to explore RNs’ perceptions on the impact of nursing shortage of nurses and their performance in providing quality care in Republic of Vanuatu in 2020.

## Methodology

### Study design and setting

A qualitative study was used to gather information using face-to-face in-depth interviews from RNs in three hospitals in Vanuatu between 4^th^ to 14^th^ September, 2020. The three hospitals were randomly selected among six hospitals that included Vila Central Hospital (VCH) in Shefa Province, Northern Provincial Hospital (NPH) in Sanma Province and Lenakel hospital in Tafea Province. In-depth interviews are very powerful methods to allow participants to express their view freely regarding their detailed personal experiences [15, 16].

### Study population and sample

All RNs in Vanuatu were considered as the study population and those who were currently working at the three selected hospitals with at least 6 months’ work experience were included in this study. Those who were not willing to participate in the study were non-respondent. A purposive sampling was used to choose study participants. The RNs were interviewed using face-to-face, in-depth interviews until data saturation is reached. A total of 25 RNs were involved in this study.

### Data collection tool

In-depth face-to-face interviews was conducted using a semi-structured open-ended questionnaire to probe elicit information from the identified participants from both the target populations. Open-ended questions aimed for participants to express their personal experience freely [17]. The interview questions developed is based on relevant literatures and research studies that will fulfill the aim and the research question of the study. Seven questions were prepared and asked during in-depth interview to enable the participants to explain or discuss their perceptions about the research topic.

The demographic information form was also used to collect demographic characteristics regarding their gender, age, marital status, education level, work station and years of experience. The interview questions were checked by 3 experts in the relevant filed and also by 3 RNs to make sure they are understandable and are in line with the research questions before conducting the interviews.

### Study procedures

Following the ethic approvals, all potential RNs in three selected hospitals were informed about the aim of study and were invited to participate. An information sheet was used to inform the participants about the purpose, procedure and nature of the study; duration of interview; the right to participate; benefits and risks of the study; notification for decline or withdrawal at any time from participating; informed consent and the interview procedure. They were informed that their information will be confidential and they are allowed to leave the study at any time. Those who met the study criteria and were willing to participate were asked to sign a consent form. An arrangement was made about the date, time and venue of the interview. A trained bi-lingual interviewer who signed a consent form was employed to conduct interviews. Participants were asked about their preferred language to do interview before the interview. Those who preferred to speak in local language were interviewed in Bislama language otherwise the English language was chosen for the interviews. All interviews were audio-taped for transcription later.

### Data management and analysis

Cross translation was applied for translating the interviews that were in Bislama to English. All the interviews were transcribed by the main researcher and were checked by the research assistant to make sure they are transcribed accurately. The data were manually analyzed using thematic analysis process to identify the final themes. Thematic analysis is a method which involves identifying, analyzing, and reporting patterns of data and is widely used for analyzing qualitative research [18]. The participants’ answers were read and re-read closely by the main researcher to divide into key words or phrases into their similar meanings and create codes. The transcribed results were later transferred to A4 paper. Then the coded data were sorted into themes and sub-themes based on the similar issues which formed the result of the study.

### Ethics approval

Before proceeding to data collection, ethic approvals were obtained from the College Health Research Ethics Committee (CHREC) in Fiji National University (FNU) and from the Research Ethics Committee in Vanuatu MoH. All participate were provided a consent form and the information sheet. The participants were informed about the purpose of the study and ensures that their identities are anonymous and the participants ‘data and any other information would be kept confidential and protected.

## Result

### Demographic characteristics of participants

Twenty-five participants were involved in the in-depth interview (12 males and 13 females). With respect of age, 14 with age range <40 and 6 of the participant with the age range from 40–49, and 5 age ≥50, and 18 of them were married. Their educational level, 21 of them had their undergraduate qualification and 4 had their highest qualification as post graduate level which includes post graduate diploma (Table 1).

**Table 1:** Demographics characteristics of participants (n = 25).

		Frequency	Percentage
Sex	Male	12	48
	Female	13	52
Age (YR)	<40	14	56
	40–49	6	24
	≥50	5	20
Marital Status	Single	2	8
	Married	18	72
	*Others	5	20
Work Experience (YR)	<10	10	40
	10–19	7	28
	≥20	8	32
Education Level (Profession)	Undergraduate	21	84
	Post graduate	4	16

*De Factor 2, Divorce 3

### Themes and sub-themes

The thematic analysis found three major themes emerging; 1) Difficult working condition, 2) Reinforcing Factors, and 3) Perceived risks. Each theme had several sub-themes (Table 2). The participants’ reflection for each theme and sub-theme are further expanded and compared with other published studies. In this section, participants are presented with a “P” and cardinal number like P1, P2.

**Table 2:** Themes and sub themes raised from the interviews.

THEMES	SUB-THEMES
Difficult working conditions	• Heavy workload
	• Lack of workforce
	• Unusual working hours
Reinforcing factors	• Lack of support
	• Lack of opportunities and advancement in nursing practice.
Perceived risks	• Stress
	• Physical and mental risk
	• Medical risk
	• Social and family risk

### Difficult working conditions

The nurses believe that the conditions where nurses’ work can have a major influence on their performance and the quality of care provided to patients include “heavy workload”, “lack of workforce” and “unusual working hours”.

#### 1. Heavy workload

All the participants (25) working in the hospitals have confirmed that workload has been a challenge when there are extremely limited nurses to manage the patients on each shift. P3 stated that shortage of nursing and workload is seen throughout the hospital wards which exceed the number of nurses working per shift.

*“Shortage of nursing is seen throughout the hospital wards and is a long-term issue where workload exceeds the number of nurses working in one shift”*.P3 (a 56-year-old female RN).

All the participants (25) also reported that the workload is increasing because of the high number of patients’ admitted. P16 compared the population in the past with the current and stated that when the population increased, diseases also increased that caused workload on nurses.

*“In the past*, *the population was less but now the population increases due to the high number of disease cases that causes more patients’ admission and more workload to us nurses”*.P16 (a 32-year-old male RN)

Some of the participants (15) reported an inadequate number of nurses working in each shift also create challenges due to workload when other nurses on sick calls or annual leave. P6 expressed the workload when only one nurse worked to cover for nurses who were on various leaves.

*“Workload is too much as most of the time only two nurses working in each shift is not enough*, *if one staff on sick leave or annual leave then we must double the shift”*. P6 (a 34-year-old male RN)

Four participants stressed the ratio of nurses to patients admitted in the hospital in Vanuatu as a huge difference which affects nurses’ performance compared to the other countries. P14 stated:

*“Uh…*. *when we look at the ratio of nurses to patients in Vanuatu which is 1*:*10 or 1*:*15 compared to other countries of which they have 1*:*4*, *there is a huge difference*. *One ward receives on average of 20 to 30 patients at one time but only 2 to 3 nurses work on one shift which is too much for one nurse to perform his or her duty effectively”*.P14 (a 33-year-old male RN)

Twenty participants have the same responses due to the nursing shortage they experienced in their workstation, that they neglected a lot of their duties and responsibilities as a registered nurse. P8 reported that the impact of shortage prevents him to perform his duties and responsibilities such as home visits and other bedside nursing care which also affects the quality of care the patients required.

*“Impact of shortage prevents me from performing some of my duties and responsibilities such as home visits and follow-up care to patients with chronic illnesses*. *Bedside nursing and wound care or wound management are also not done regularly*, *which can have a great impact on patients’ health”*. P8 (a 43-year-old male RN)

#### 2. Lack of workforce

Increased workload compared to less number of nurses working in the hospitals causes nurses’ physical exhaustion leading to job dissatisfaction as expressed by all 25 participants. P11 expressed the result of lack of workforce to his well-being.

*“Workload is too much in the hospital wards and we cannot do all our work at one time……I normally experienced tiredness and exhaustion and not interested to work due to incomplete jobs seen each day”*. P11 (a 37-year-old male RN)

Thirteen of the participants responded that the increased workload does not correspond with the number of nursing staff in the health facilities especially with increased number of patients admitted and less number of nurses working. P8 stated that the number of workforce does not match with the number of workload from increased admission.

*“Few nurses do not match with the increased workload today*. *For example*, *increased number of admissions with only 2 staff working per shift is a great challenge to us”*.P8 (a 43-year-old male RN)

Other participant added:

*“Shortage in my ward with only 2 nurses in one shift is not enough compared to the number of patients admitted especially when we have the critical patients that need close supervision in the ward”*. P22 (a 53-year-old female RN).

Furthermore, eight participants stated that training and enrolment have significant effects to the shortage on the nursing workforce due to a single nursing college in the country with limited number of student nurses’ enrolment. P6 said that lack of workforce is due to inadequate enrolment from the nursing college each year.

“*One nursing college is not enough to train more nurses to have an adequate number of nurses in the workforce*. *Furthermore*, *the decreased number of intakes to only 30 per year is not enough”*. P6 (a 34-year-old male RN)

Conversely, seven participants stated that lack of nursing workforce is due to irregular nursing enrollment in the nursing college in the past.

*“The reason for having a shortage of nurses frequently is due to uhm……no regular nursing intake from the VCNE each year*. *In the past 15 years*, *nursing college always have regular intakes each year even if the number of intakes is less*, *we still have continuous graduation of nurses each year with a good supply of nurses in the hospital to work and provide care*. *Nowadays*, *the intake occur every 2 or 3 years*. P20 (a 33-year-old female RN)

Few of the participants (4), reported that the other reasons for lack of workforce is nurse turnover. P24 stated that the workforce is affected especially when nurses leave their profession and look for other jobs elsewhere due to too much pressure from work.

*“Workforce is affected when nurses leave their profession and look for other jobs elsewhere*. *They left due to too much work load and not enough time to rest”*. P24 (a 42-year-old female RN).

#### 3. Unusual working hours

Working long shift hours up to 12 to 16 hours or double the shift due to not enough staff to do shift work especially when staff on sick leave or on annual leave causes physical and emotional exhaustion and also affects quality patients’ care. P21 expressed the reasons for long hours shift and its impact to the nurses and to the patient.

*“Most of the time we spend long shift hours of work e*.*g*. *12 to 16 hours or we double the shift due to not enough staff in the ward to do shift work when we don’t have enough staff and when staff are on sick leave*. *It is so tiring and causes a lot of stress to most of us who work long hours which also affect the quality care provided to patient”*.P21 (a 42-year-old female RN)

Fifteen of the participants who normally work shift stated that they used to work double shift especially during the night where only few nurses were working. P17, an experienced nurse expressed that double shifts especially at night is common in the hospital wards when nurses on duty unexpectedly on sick leave which significantly affect the nurses’ physical well-being.

*“Double shift is a common practice in the wards especially when there are not enough nurses to work or when a working colleague is on sick leave*. *This causes much stress to us nurses due to tiredness”*. P17 (a 64-year-old female RN)

Five senior nurses responded that occasionally they work 24 hours to assist nurses in the ward when more critical patients are admitted or during an epidemic. P3 stated that as a senior in charge nurse, they committed to work 24 hours when lack of nurses to take care of increased patient admission

*“It is our duty as senior nurses to assist the nurses in the wards when more critical patients are admitted or during disease outbreak and work for 24 hours*. *It is quite tiring but we have no choice because it is part of our responsibilities”*. P3 (a 56-year-old male RN)

Three participants responded that during natural disasters, where a lot of nurses are unable to attend work and more patients admitted, they have to work extra hours during the day and during the night. P22 expressed her experience during natural disasters where she has to work on unusual hours to care for the casualties and assist nurses in the wards.

*I have experienced spending all day and night for one whole week during tropical cyclones to look after patients as more nurses were unable to come to work”*. P22 (a 53-year-old female RN).

### Reinforcing factors

The nurses quoted during the interviews that “lack of support” and “lack of development opportunities and advancement in nursing practice” were reasons for low motivations in their performance and job retention.

#### 1. Lack of support

Most respondents (13) reported that lack of support from the leaders causes low working morale and low motivation. P15 stated that the leaders in the hospital management haven’t provide much support to the nurses.

*“We always confront our nursing managers or clinical supervisors concerning problems in our work place such as poor working equipment needing replacement and poor working environment but they always give excuses and no action taken seriously which affects our morale of work*….” P15 (a 35-year-old male RN)

Another participant added:

*“We hardly see the managers or supervisors doing regular visitation to support nursing staff and to assess nurses work performance*, *this causes low staff motivation”*. P14 (a 33-year-old male RN)

All nurses (25) responded that lack of family support is common due to working overtime and coming home late from work. One participant (P21) reported that they don’t receive any support from the family especially when they came home late from work

*“When I came home very late from work my family got angry with me*. *I don’t receive any support from my family*. *They even forced me to quit my job due to coming home late from work every day”*. P21 (a 42-year-old female RN)

Another participant added:

*“Even my family don’t want to give me food due to frustration of continuously coming home late from work*.*”* P16 (a 32-year-old male RN)

Most of the participants (15) have expressed their frustration due to lack of financial support from the MoH especially special allowance for working overtime and others. P1 stressed that she has been working for more than 20 years but she hasn’t received any financial support concerning their overtime package or other allowance or incentives apart from their normal wages which affect their motivation to perform duty effectively.

*“I work for many years but I don’t receive any financial support from the health authorities apart from my little salary regarding extra responsibility allowances or overtime allowances or any incentives”*. P1 (a 56-year-old female RN)

Other participant added:

*“Even our working status is on contract bases for so long due to positions not budgeted for which affects our benefits and job insecurity”*. P11 (a 37-year-old male RN)

#### 2. Lack of development opportunities and advancement in nursing practice

All participants (25) stated that lack of development opportunities to advance in nursing practices and career pathways are common problems that cause disappointment within the working environment. P24 expressed her disappointment that she works for quite a long time in the hospital but chances to advance in her knowledge is very slim and don’t have the opportunity to expand her knowledge and skills in nursing practice.

*“I am very disappointed because I worked in the hospital for many years doing the same routine job as usual and I still remain the same usual nurse…*. *I don’t receive any promotion because I don’t have any opportunities to advance in knowledge and skills in nursing practices”*. P24 (a 42-year-old female RN)

Another participant added:

*“I haven’t seen any effective career pathway for nurses developed by managers for further trainings to upgrade nurses’ knowledge and skills for advancement in our clinical practice”*. P4 (a 34-year-old male RN)

All the participants stated that most of them don’t have any chances for professional development. P18 responded that most nurses perused their training from the Vanuatu nursing college with a diploma level and haven’t had any chances to upgrade to a higher level of qualification.

“*Most of us nurses graduated from the nursing college with a diploma of nursing but we don’t have changes to upgrade to a higher level of qualification or to up skill our-selves”*. P18 (a 30-year-old female RN)

Another participant added:

*“Our skills in nursing practice need to be upgraded in order for us to advance with our clinical practices*. *It is very good to have regular in-service training but it never happens on regular bases*, *in order to keep us updated with our nursing practice skills*”. P2 (a 34-year-old male RN)

Other nurses reported that specialty training is also necessary to up skill nurses and advance in their clinical practice in the speciality area but only few nurses had given the chances in the past to attend those training.

*“Vanuatu needs more specialized nurses to provide quality care to different types of patients however*, *only few nurses had been given the chances to take up those training which is still needed for more nurses to take specialize training to provide effective and quality care needed”*. P3 (a 56-year-old female RN)

### Perceived risk

The nurses quoted during interviews that “stress” and “physical and medical risks” were reasons that affect nurses and increased the chances to quit their profession.

#### 1. Stress

Majority of the participants (20) have worked in the hospital for more than 5 years and reported that they have experienced the impact of shortage of nursing personally. P5 reported that stress causes a major effect on nurses due to workload and also threatens her job.

*“I experienced tiredness*, *stress and not satisfied with my job each day due to work overload*. *I normally go home late due to long hours of work and no time for my family which affects my family relationship*. *Even my family asked me to look for another health facility to work which has less workload”*. P5 (a 31-year-old female RN)

Four of the participants stressed the effects of work overload and overtime due to nursing shortage causes stress and frustration and violence at home.

*“Work overload and work for long hours causes a lot of stress and frustration where I don’t have enough rest*, *no time to relax*, *and not enough quality time for my family which causes frustration and violence in my home”*. P23 (a 53-year-old female RN)

*O*ther participants (12) added:

*“Stress is the result of tiredness and not enough rest especially when the ward is full and less nurses working and you have to double the shift”*. P12 (a 40-year-old female RN)

#### 2. Physical and mental risks

Some participants (6) stated that work overload and work for long hours causes more physical and medical risks

*“Shortage of nurses affects our physical body very badly*. *We experienced back pain and back injury for trolleying patients to the theatre and to other diagnostic units……and we felt tired and cannot provide the best quality nursing care to our patients”*. P20 (a 33-year-old female RN)

Other physical risks which was reported by all participants (25) is when they don’t have enough time to rest and eat or drink due to too much work load and limited nurses. P24 expressed that they don’t have enough time to rest and eat during busy times which affect her physical body and her health.

*“Most of the time our ward is busy and those times I don’t have enough time to rest and eat or even drink which affects my physical health”*. P24 (a 42-year-old female RN)

Workload with only few nurses causes a lot of medical risks on nurses’ health and clinical performance which leads to early retirement or were granted early retirement due to medical reasons. One participant stated:

*“A lot of nurses in our hospital leave their job and most of them were granted early retirement due to medical health reasons which prevent them to continue with their job”*. P11 (a 37-year-old-female)

Another participant added:

*“I worked almost 20 years now and I have medical issues which affect both my lower extremities and I have requested to take my early retirement because I won’t be able to work with the current health conditions*. *My health conditions will not only affect my well-being but will also affect my clinical nursing performance”*. P7 (40-year-old-female RN)

Most nurses (15) reported that high job demands increase physical and mental health problems. P9 mentioned the impact of stress to physical and mental problem on nurses

*“Stress affects our mental health when we are exhausted due to work overload which prevents us to think properly which also increases the chances to make mistakes”*. P9 (a 56-year-old female RN)

Another participant added:

*“When we have too many patients and lack of skills especially for us inexperienced nurses*, *it affects us psychologically as well which can affect our performance”*. P5 (a 31-year-old male RN)

#### 3. Medical risk

One of the respondents stated that medical errors are one of the common risks that occur due to stress from working long hours or work overload.

*“I have experienced the result of stress that causes high chances of errors in our work station which threaten the lives of the patient*. *Some prevented errors are the result of work overload and long hours of work which prevent nurses from perform their duties effectively and increase the chances to make mistakes”*. P2 (a 34-year-old male RN)

Four participants reported that medical errors were seen in their work station due to physical and psychological stress where they gave incorrect medication to the patients.

*“Few times I gave incorrect medication to patients because I can’t think properly due to tiredness and exhaustion or sometimes I gave the correct medication but I don’t explain it well to the patient especially the dose*, *time and route of administration”*. P4 (a 34-year-old male)

Another participant added:

*“Most of the time due to frustration and too much workload I don’t practice infection control rules and regulations which cause more medical risk to my patients”*. P11 (a 37-year-old male RN)

#### 4. Social and family risk

Nurses experienced social and family risks when they have high volume of pressure and when patients are not receiving services immediately, they cause mischief to nurses and their families. P12 expressed his fear when patient and relatives were frustrated due to patients’ not receiving care or service immediately and threaten her family.

*“I experienced most times especially when we have less nurses working in one shift in the emergency department when I and even my family were threatened when patients’ relatives got angry with me for not attending to them immediately or not treating them well as expected*. *Sometimes they threatened me and my family as well”*. P12 (a 40-year-old female RN)

Another participant added:

*“Occasionally I get frustrated from work due to pressure and when I bring frustration to my home*, *it causes domestic violence in my home*. *This causes much risk to my family”*. P6 (a 34-year-old male RN)

## Discussion

Prompted by the findings from the RNs in Vanuatu on the nursing shortage, it impacted the health service delivery throughout the Vanuatu population [14]. Although the Vanuatu MoH has been implementing strategies in the past to address the issues, the shortage of nursing is still evident with the current nursing workforce shortage of more than 400 where Vanuatu MoH is still unable to fill the shortage gaps [14]. The current study findings have reported the impact of nursing shortage on the nurses and their performance in providing quality care.

### Difficult working conditions

The working conditions for nurses have major influence on the nurse’s performance and the quality of care provided to patients due to job dissatisfaction. The findings emerged with the condition which includes workload due to high patients’ admission, lack of workforce and unusual working hours. Several studies have shown that job dissatisfaction always emerged along with poor working conditions due to workload and lack of workforce [19, 20].

It is obvious that the workload in the health facilities within the MoH health system has been a long-term issue and become a challenge when few or limited number of nurses who care for the large number of patients admitted, and workload exceeds the number of nurses working in each shift. The maximum number of nurses working per shift is 2 to 3 nurses according to the findings, which is not effective to provide a quality care needed for nurses and patient’s safety. Although the managers within the hospital setting are aware of the workload issues, they have no better solutions to address the workforce shortage as it become a major challenge across the country that needs effective planning and policy directions from the policy makers at the government level. Studies stressed that work load is becoming a major factor when there are inadequate number of nurses working compared to the demand [21, 22]. Other studies from other developed countries also reported that inadequate policy direction and planning has huge impact on nursing population including nurses’ workload [5, 23]. The difference is that our study participants have experienced shortage and its impact while working in the hospital and might have limited knowledge about the policy and planning direction of Vanuatu MoH.

With few number of nursing staff compared to high workload, causes a lot of pressure and physical exhaustion to nurses. There are factors that contributed to lack of workforce identified by participants who include low student nurse enrolment or irregular training provided by the nursing college. Although the nursing college enrolled nurses continuously for the last 30 years, the number of output is so limited and does not match with the increased demand. Other health leaders also supported the fact that low enrolment in the nursing college is becoming obvious when looking at the current increase number of aging population of nurse within the MoH. Studies from other countries stated that low enrolment have significantly contributed to lack of workforce which affect nursing and their profession in the future [4, 24]. The shortage were identified by the participants from the low number of nurses distributed and work in each of the hospital.

In this study it was found that most of the nurses working in the hospital had experienced long shift hours up to 12 to 16 hours or double the shift due to not enough staff to do shift work or when other nursing staff on sick calls or annual leave. The nurses stated that long working hours is very stressful which affects their work performance and as well as their social and family relationship. The nursing managers and senior clinical supervisors aware that nurses normally work on unusual hours when not enough staff to do shift work, and have noticed moral distress on nurses which affects nurses’ motivation to perform the job effectively. In other industrialized countries, one third of the nursing workforce has irregular or unusual working hours which significantly affects the nurses’ health and patient outcome [25]. Furthermore, pressure of working long hours contributed to nurses leaving their profession from job dissatisfaction and poor working environment. Study have shown that nurses leave their job due dissatisfaction with working condition in a stressed environment such as irregular working hours [26, 27].

### Reinforcing factors

Findings shows that lack of support and lack of development opportunities and advancement in nursing practice were reasons for low motivations which affects nurses’ performance.

Most nurses reported that lack of support from the managers and supervisors causes low working morale and low motivation to perform duties effectively. Although the nursing managers and senior clinical staff are experienced in their position, nurses still haven’t received full support for the leaders. This includes no regular visits and no actions to nursing staff complaints or grievance. It is evident due to areas that yet to be resolved and need urgent actions from the managers. Studies show that nurses needed attention from the managers and supervisors to identify areas that needs urgent or serious attention or early detection of any problems that might occur among nurses and their work performance [20, 28].

Most of the nurses stated that lack of development opportunities to advance in nursing practices is one of the common issues that create disappointment on nursing staff within their working environments. Nurses believed that when opportunities to advance is left too long or no attention from their superiors, it causes low motivation that leads to low performance that will certainly allow nurses to leave their job. Although the HR at the national level develop career pathway for nurses, most nurses are not given any chances to advance in their profession or capacity building as part of their professional development, which is also reflected on the level of Education on demographic information where the highest level for most nurses is diploma of nursing. A study in Iran has shown that lack of opportunities to advance in nursing and lack of professional vision towards nursing, cause discrimination among nurses and dissatisfaction which causes nurses intended to leave their profession [21]. According to the RNs personal characteristics, more than 50% have completed undergraduate studies with diploma of nursing as their highest level of nursing which reflect lack of professional vision to upgrade nurses to higher level which might results to low motivation in the workplace.

### Perceived risk

The findings perceived that stress and medical risks impact nurses that increase the chances to quit nursing profession.

Stress has major effect on nurses not only with physical exhausted but also has an effect on social and family relationship. Stress affected nurses due to workload and overwork which significantly affect the quality of care provided to patients as well. Majority of the RNs who work in the hospital might experience the impact of shortage of nursing personally because they have worked for more than five years. If stress was managed promptly, it will prevent burnout, job satisfaction and improve patients’ quality care. Although studies have shown that stress affect all nurses due to worldwide nursing shortage, the nursing managers and leaders in Vanuatu, who have in contact with nurses regularly must have better understanding of stress and its relationship and also its symptoms in order to manage stress effectively [29, 30]. It is important for Vanuatu MoH to adopt stress management process by other countries in order to identify and management stress among nurses effectively.

Findings have shown that work overload and long hours’ work causes serious threats to nurse’s physical health. Furthermore, finding shows that nurses experienced injuries and other medical conditions while performing service. Furthermore, majority of the RNs have been granted early retirement due to medical reasons that might be due to work overload or poor working conditions. The challenges of having nurses gone on early retirement is when not enough nurses for replacement, however nurses with medical reasons need to leave their profession as they will negatively impact patient’s care and also their well-being. Studies supported that mental and physical health of nurses has significant effects on the quality of care provided to the patient [30, 31]. On the other hand, other studies supported that senior nurses leave their profession before their retirement age due to medical reason and is necessary for patients’ safety to decrease mortality [32, 33].

Furthermore, stress associated with nursing shortage has a significant impact on patients’ care in the hospital which causes much health risks and increase the risk of medical errors and lack of quality care up to a required standard. Findings show that, Vanuatu nurses were able to work under pressure, but medical errors can still be experienced at the workplace. Studies confirmed that medical errors are associated with nurses’ psychological stress and other health risks due to work overload [8, 31].

### Study strengths

The study is a high quality study and the first study that was conducted in the Republic of Vanuatu among the registered nurses. The study rigors was followed from conducting the study, data collection and data analysis. The study will benefit the Vanuatu ministry of health by enabling the policy makers to refine and develop relevant policies to address and strengthen the nursing workforce to meet the demand and improve delivery of quality health services to all individuals in both urban and rural settings.

### Study limitations

There were some logistic limitations in terms of conducting interviews or reach the study participants easily due to was unable to Covid-19 pandemic. It was not possible to study other hospitals in Vanuatu to extract more information due to time limitation.

## Conclusion

This study has identified many key factors that contributed to the shortage current nursing workforce and the impact it has on RNs which needs to be addressed promptly to resolve the shortage of nursing workforce Vanuatu in the coming years. Broad themes and sub-themes were identified which highlighted the impact of nursing shortage to RNs and the effects on their performance. The studies showed that stress or moral distress from work overload and lengthy hours shift impact the nurses’ physical, psychological, social, and family relationship.

The recommendation to assist the Vanuatu government through the Ministry of health to address chronic shortage of nurses is, the government should invest on establishing a much bigger nursing college to increase its yearly intake in nursing, to have interim plan to address the current shortage of nurses and review the whole nursing situation and nurses distribution, and to promote nursing in all the secondary levels of education.
